# Web-based GIS: the vector-borne disease airline importation risk (VBD-AIR) tool

**DOI:** 10.1186/1476-072X-11-33

**Published:** 2012-08-14

**Authors:** Zhuojie Huang, Anirrudha Das, Youliang Qiu, Andrew J Tatem

**Affiliations:** 1 Department of Geography, University of Florida, Gainesville, FL, USA; 2 Emerging Pathogen Institute, University of Florida, Gainesville, FL, USA; 3 Fogarty International Center, National Institutes of Health, Bethesda, USA

**Keywords:** Infectious disease, Air transport network, Imported disease, Web GIS, Malaria, Dengue, Yellow fever, Chikungunya, Mosquito

## Abstract

**Background:**

Over the past century, the size and complexity of the air travel network has increased dramatically. Nowadays, there are 29.6 million scheduled flights per year and around 2.7 billion passengers are transported annually. The rapid expansion of the network increasingly connects regions of endemic vector-borne disease with the rest of the world, resulting in challenges to health systems worldwide in terms of vector-borne pathogen importation and disease vector invasion events. Here we describe the development of a user-friendly Web-based GIS tool: the Vector-Borne Disease Airline Importation Risk Tool (VBD-AIR), to help better define the roles of airports and airlines in the transmission and spread of vector-borne diseases.

**Methods:**

Spatial datasets on modeled global disease and vector distributions, as well as climatic and air network traffic data were assembled. These were combined to derive relative risk metrics via air travel for imported infections, imported vectors and onward transmission, and incorporated into a three-tier server architecture in a Model-View-Controller framework with distributed GIS components. A user-friendly web-portal was built that enables dynamic querying of the spatial databases to provide relevant information.

**Results:**

The VBD-AIR tool constructed enables the user to explore the interrelationships among modeled global distributions of vector-borne infectious diseases (malaria. dengue, yellow fever and chikungunya) and international air service routes to quantify seasonally changing risks of vector and vector-borne disease importation and spread by air travel, forming an evidence base to help plan mitigation strategies. The VBD-AIR tool is available at http://www.vbd-air.com.

**Conclusions:**

VBD-AIR supports a data flow that generates analytical results from disparate but complementary datasets into an organized cartographical presentation on a web map for the assessment of vector-borne disease movements on the air travel network. The framework built provides a flexible and robust informatics infrastructure by separating the modules of functionality through an ontological model for vector-borne disease. The VBD‒AIR tool is designed as an evidence base for visualizing the risks of vector-borne disease by air travel for a wide range of users, including planners and decisions makers based in state and local government, and in particular, those at international and domestic airports tasked with planning for health risks and allocating limited resources.

## Background

Over the past century, the size and the complexity of air travel network has increased substantially. Nowadays, there are 29.6 million scheduled departure flights per year and around 2.7 billion passengers are transported annually [1]. Air travel has changed the epidemiological landscape of the world, providing routes from one side of the Earth to the other that can be traversed by an infected person in significantly shorter times than the incubation period of the majority of infectious diseases. This epidemiological impact has led to a rethink in global disease management [2], with pandemic control being less reliant on conventional spatial barriers as the global air network continues to expand. Today, vector-borne diseases and vectors are moving between different regions at unprecedented rates, resulting in adverse ecological, economic and human health consequences [3-5]. Reducing these problems requires examination of how humans facilitate the movement and establishment of diseases and their vectors in new areas. Moreover, the speed of air travel has meant that rapid reporting and surveillance now play an important role in preventing the spread of diseases. Finally, the cost of surveillance makes sampling design and the development of cost effective monitoring and testing approaches vital in effective early-warning systems [6]. While work on these factors is becoming more sophisticated for directly-transmitted infections [7-9], our understanding of the role of air travel in global vector-borne disease epidemiology remains relatively incomplete [3].

Substantial evidence exists that documents examples of both vector-borne diseases and the vectors that carry them being transported between distant locations via air travel [10,11]. The global air network enables many of the world’s most isolated and diverse ecosystems to become connected and aids the movement of organisms, including disease vectors, to new habitats where they can become damaging invasive species, economically and health-wise [4,12,13]. Mosquitoes can survive moderately high atmospheric pressures aboard aircraft [14] and can be transported alive between international destinations, even in wheel bays [15]. However, air travel likely plays a much more significant role in moving the vector-borne disease (via infected passengers) than in moving the vector. It provides rapid and wide-reaching connections between outbreaks or high levels of endemicity and susceptible vector populations elsewhere in the world. Up to 8 % of travellers to the developing world become ill enough to seek healthcare upon returning home, with a significant proportion of these suffering from vector-borne infections [11]. Around 10,000 cases of imported malaria to high income countries are reported each year, but the true figure may be over 25,000 [16]. With imported vector-borne infections placing a financial and operational burden on health systems in non-endemic countries (e.g. [17]), as well as the risk of onward transmission and even establishment, the development of tools for quantifying the spatiotemporal risks of importation of both vector-borne diseases and the vectors that carry them can be valuable for assessing and guiding the allocation of limited control and surveillance resources.

Recent efforts in the field of global mapping of vector-borne diseases (e.g. [18,19]) and the vectors that carry them (e.g. [20-22]), generally through using sample data of known disease or vector presence in combination with environmental covariates, now provide strong evidence bases for determining vector-borne disease risks across the world. The linkage of these disease and vector distribution maps with air travel network data offers great potential for infection importation risk assessment and the modelling of vector-borne disease spread. Such distribution maps, however, represent static pictures of relatively long term (>1 year) disease prevalence and vector presence. If importation and onward spread risks are to be accurately quantified, the substantial climate-driven seasonal fluctuations in disease risk and vector densities need to be accounted for [23]. If a vector or an infected individual arrives in a new location via air travel, the risk of the vector establishing or the infection being passed on to local vector populations is often dependent upon the month of arrival. The arrival of an individual infected in a mosquito-borne disease outbreak occurring in January in the southern hemisphere (e.g. Sao Paulo) into a city in the northern hemisphere (e.g. New York) will present little risk of onward transmission, due to the cold January climate being not conducive for mosquito activity. However, a similar arrival from e.g. India (where climatic conditions may be suitable for year-round transmission) in July presents a much greater risk [23]. By utilizing gridded climate data to measure climatic similarity between origin and destination locations with known presence of a suitable vector, and adjusting for flight passenger numbers as an additional measure of risk, these factors can be accounted for [4,24].

Here we introduce the Vector-Borne Disease Airline Importation Risk tool (VBD-AIR; http://www.vbd-air.com), which brings together global vector-borne disease and vector distribution maps, climate data and air network traffic information to inform on the spatiotemporal risks of disease and vector importation. The VBD-AIR tool takes the form of an interactive online interface and is targeted at users with interests in specific airports or regions, and the risks to those locations of vector-borne disease importation and onward spread, or exotic vector importation and establishment.

### Data

VBD-AIR utilizes an entity-relationship model to integrate data sources from airport locations and air routes, disease and vector distributions, global climate data, and global land-based travel time data. All of these datasets are described below.

### Air travel data

**Figure 1:**
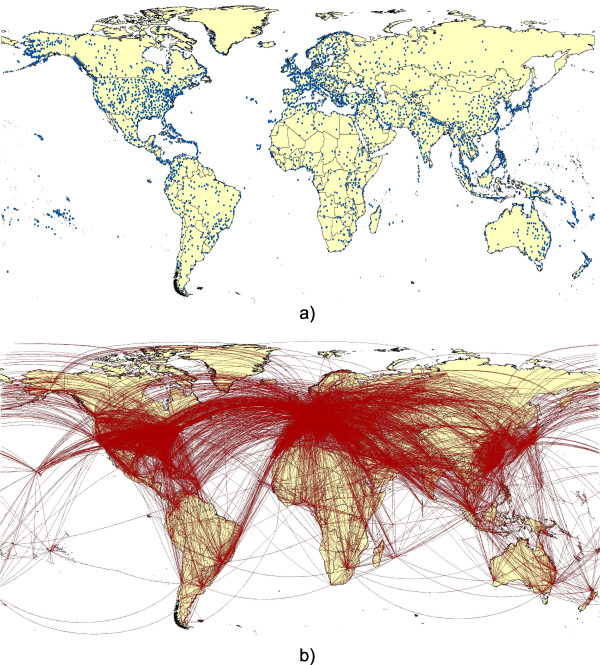
**2011 Air network data used in VBD-AIR.** (**a**) The location of the 3,632 airports across the world; (**b**) the flight routes for 2011 captured within the VBD-AIR tool.

Information on a total of 3,632 airports across the world, together with their coordinate locations was obtained using Flightstats (http://www.flightstats.com) and is mapped in Figure1a. Information on the airport name, IATA code, city and country are all stored in the VBD-AIR database that was constructed for the tool (see methods). Flight schedule and seat capacity data for 2010 and 2011 were purchased from OAG (Official Airline Guide, http://www.OAG.com). These included information on origin and destination airports, flight distances, and passenger capacity by month of each year. All routes used in the tool are mapped in Figure1b.

### Disease distribution datasets

**Figure 2:**
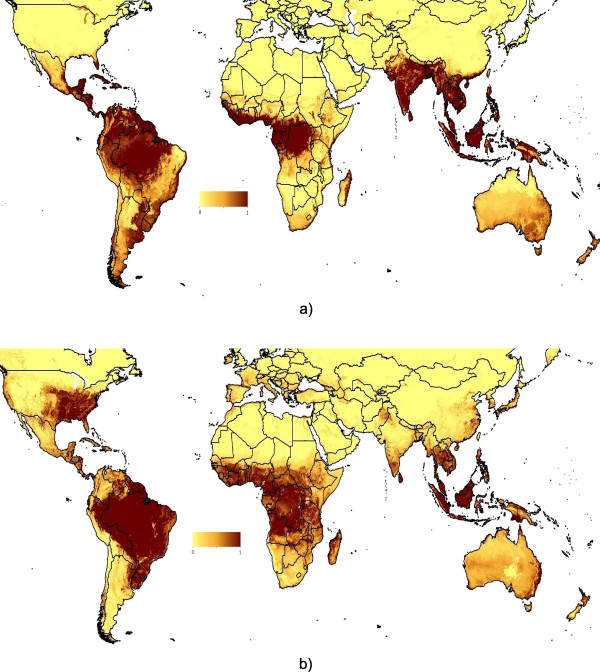
**Example disease and vector distribution maps from the VBD-AIR tool.** The predicted distribution of (**a**) chikungunya outbreak risk based on geolocated data on recorded outbreaks since 2008 combined with satellite-derived environmental covariates within a boosted regression tree species distribution prediction model. The color scale shows predicted unsuitable to suitable conditions for outbreaks as a continuous scale from yellow to dark blue. (**b**) climatic and environmental suitability for *Aedes albopictus* presence based on field survey data combined with satellite-derived environmental covariates within a boosted regression tree species distribution prediction model. The colour scale shows predicted unsuitable to suitable conditions as a continuous scale from yellow to dark blue.

The current version of VBD-AIR at the time of writing focuses on four vector-borne diseases and their related vectors, chosen due to the availability of spatially-referenced data for map production and rates of importation by air travel: Malaria (*Plasmodium falciparum* and *P. vivax*), dengue, yellow fever and chikungunya, all transmitted by mosquitoes. The methods behind the construction of each of these datasets are described briefly here, with an example map that is shown in Figure2a, and the remaining output maps and mapping process presented in Additional file 1.

*Plasmodium falciparum* is a protozoan parasite, one of the species of *Plasmodium* that cause malaria in humans. *P. falciparum* is the most dangerous of these infections as *P. falciparum* (or malignant) malaria has the highest rates of complications and mortality, while *P. vivax* is the most frequent and widely distributed cause of recurring (tertian) malaria. Non-endemic countries often see many cases of imported malaria each year through travellers or returning migrants. The geographical distribution of predicted *P. falciparum* malaria endemicity in 2010 was obtained from the Malaria Atlas Project (http://www.map.ox.ac.uk) and the methods behind the construction of the map are presented in Gething *et al.*[18]. In brief, 22,212 community prevalence surveys were used in combination with model-based geostatistical methods to map the prevalence of *P. falciparum* globally within limits of transmission estimated by annual parasite incidence and satellite covariate data [25]. The mapping and modeling framework for *P. vivax* is presently not as advanced as for *P. falciparum*, however, the limits of transmission and stable/unstable endemicity have been mapped [26], and this dataset was used within VBD-AIR to define areas of *P. vivax* malaria endemicity.

Dengue fever is an infectious tropical disease caused by the dengue virus. The incidence of dengue fever has increased dramatically since the 1960s, with around 50–100 million people infected yearly, and imported cases through air travel to non-endemic regions are on the rise [27]. Global dengue distribution was mapped in three different ways: (i) a map of environmental 'suitability' for transmission, (ii) the same map, but only with countries/regions of known recent transmission shown, and (iii) a map of environmental similarity to recent outbreaks. For construction of the first map, thousands of geographically-located data points on dengue transmission over the past decade were combined with climatic and environmental covariates within a boosted regression tree mapping tool [28], following the approaches of Sinka *et al.*[15-17,23], to produce a global map of suitability for dengue transmission (Additional file 1). To refine this map to be focused solely on regions of recent confirmed transmission, the second dengue map was constructed with dataset masked so that only regions cited by the CDC Yellow Book (http://wwwnc.cdc.gov/travel/yellowbook/2012/chapter-3-infectious-diseases-related-to-travel/dengue-fever-and-dengue-hemorrhagic-fever.htm) as having transmission in 2010 were left (Additional file 1). Finally, to produce an alternative, more contemporary dataset focused on regional suitability for significant outbreaks, geographical data on outbreaks occurring since 2008 were extracted from Healthmap (http://www.healthmap.org) and again combined with climatic and environmental covariates in a boosted regression tree tool [28] to map predicted dengue fever outbreak risk (Additional file 1).

Yellow fever is an acute viral hemorrhagic disease [29]. The yellow fever virus is transmitted by *Aedes aegypti* (and other species) and is found in tropical and subtropical areas in South America and Africa, but not in Asia. Since the 1980s, the number of cases of yellow fever has been increasing, making it a re-emerging disease, and case numbers imported through air travel to non-endemic areas have been rising [29]. Global yellow fever distribution was mapped in two different ways: (i) a map of environmental suitability for transmission and (ii) a map of environmental similarity to recent outbreaks. Hundreds of geographically-located data points on yellow fever occurrence over the past twenty years were combined with climatic and environmental covariates within a discriminant analysis mapping framework to produce a global map of suitability for yellow fever transmission. The datasets and methods used are described in Rogers *et al.*[19]. The risk map of environmental similarity to recent outbreaks was produced using the same methods as for the dengue outbreak-similarity map described above, but using yellow fever outbreak data from Healthmap (Additional file 1).

Chikungunya virus is an insect-borne virus, of the genus alphavirus that is transmitted to humans by virus-carrying *Aedes* mosquitoes, principally *Ae.aegypti* and *Ae.albopictus*. Large sporadic outbreaks have occurred since around 2005, with spread associated with both the movement of infected air travelers and the spread of the range of *Ae. albopictus*[30]. An outbreak risk map was produced using the same methods as outlined above for the dengue outbreak-similarity map, but using chikungunya outbreak data since 2008 from Healthmap (Figure2a).

### Vector distribution datasets

VBD-AIR includes three global vector distribution maps: *Aedes aegypti, Aedes albopictus* and the dominant *Anopheles* vectors of malaria. An example map is shown in Figure2b for *Ae. albopictus* and the remaining maps are shown in Additional file 1, with the mapping process described briefly here. More complete details on the mapping process are provided in the VBD-AIR user guide, available through the online tool (http://www.vbd-air.com).

The yellow fever mosquito, *Aedes aegypti* is a mosquito that can spread dengue fever, chikungunya and yellow fever viruses, as well as other diseases. The mosquito originated in Africa but is now found in tropical and subtropical regions throughout the world. Hundreds of geographically-located data points on field-caught *Ae. aegypti* occurrence over the past 10 years [31,32] were combined with climatic and environmental covariates within a boosted regression tree mapping framework [20-22,28] following the approaches of Sinka *et al.*[15-17,23], to produce a global map of predicted *Ae. aegypti* presence (Additional file 1).

*Ae. albopictus* is of medical and public health concern because it has been shown in the laboratory to be a highly efficient vector of 22 arboviruses, including dengue, yellow fever, and West Nile fever viruses [33]. In the wild, however, its efficiency as a vector appears to be generally low, although it has been implicated in recent dengue fever and chikungunya outbreaks in the absence of the principal vector, *Ae. aegypti*. From its Old World East Asian distribution reported in 1930, *Ae. albopictus* expanded its range first to the Pacific Islands and then, within the last 20 years, to other countries in both the Old World and the New World, principally through ship-borne transportation of eggs and larvae in tires [4,33]. Here, hundreds of geographically-located data points on field-caught *Ae. albopictus* occurrence over the past 10 years [31,32,34] were again combined with climatic and environmental covariates within a boosted regression tree mapping framework [20-22,28] to produce a global map of predicted *Ae. albopictus* presence (Figure2b).

*Anopheles* is a genus of mosquito. There are approximately 460 recognized species: while over 100 can transmit human malaria, only 30–40 commonly transmit parasites of the genus *Plasmodium*, which cause malaria in humans in endemic areas. *Anopheles gambiae* is one of the best known, because of its predominant role in the transmission of the most dangerous malaria parasite species – *Plasmodium falciparum.* Thousands of geographically-referenced data points on the presence of the dominant *Anopheles* vectors of malaria have been gathered and used, in combination with environmental covariates, expert opinion maps and regression tree tools, to produce global maps of *Anopheles* distributions by the Malaria Atlas Project (http://www.map.ox.ac.uk) [20-22,35]. Here, these maps were combined to produce a global map of dominant malaria-vector presence (Additional file 1).

### Climate and other datasets

The principal climatic constraints to vector survival (mosquitoes in this case), development and the vector-borne disease life cycle within them are temperature, rainfall and humidity. Monthly gridded global data on each of these were obtained from the CRU CL 2.0 gridded climatology datasets (http://www.cru.uea.ac.uk/cru/data/hrg/) [36]. The climate information from these gridded data was extracted for the locations of each of the airports.

A dataset depicting travel time to the nearest major settlement (with population size > 50,000) was obtained (further details here: http://bioval.jrc.ec.europa.eu/products/gam/index.htm), to provide contextual information on (i) airport disease accessibility at the origin and (ii) the potential ease of disease spread upon arrival. The risk of a disease being imported to a new location should not only be quantified by the level of predicted risk at the location of the airport, since travellers often travel long distances to get to an airport, often coming from more rural regions where disease risk may be higher. Therefore, the land-based travel-time dataset described above was used in combination with each disease distribution map to extract the maximum predicted disease risk within two hours and fifty kilometers of each airport (see Additional file 1 for more detail). There exists no globally comprehensive information on travel distances to airports, thus we used this simple assumption here. The maximum level of disease risk within these travel times and distances were assigned to each airport in all tool calculations.

## Methods

VBD-AIR is designed to be a flexible tool that combines multiple geospatial datasets to inform on the relative risks between differing airports, flight routes, times of year, diseases, and their vectors, in promoting the movement of passengers infected by vector-borne diseases and the vectors that spread these diseases. In general, the tool relies on the assumptions that the levels of imported vector and vector-borne disease risk via air travel are related to (i) the presence of flight routes connecting to endemic regions (promoting the movement of people, pathogens and vectors), (ii) the level of traffic between origin and destination (increasing the probability of infected passenger and vector carriage), and (iii) the monthly climatic similarity between origin and destination (since vector activity is required at both locations to firstly provide infected passengers, and secondly prompt onward transmission or vector establishment at the selected destination).

### Combing datasets and creating risk metrics

VBD-AIR utilizes an entity-relationship model to integrate the data sources from airport locations and air routes, disease and vector distributions, global climate data, and the global land-based travel time data. VBD-AIR can be identified as a “produser” for knowledge dissemination between authoritative data producers and expert users, as it consumes data from a spatial data infrastructure and produces analytical output [37]. To generate attributes for each airport from the spatial datasets, airports were setup as the reporting agents and implemented with an object-oriented notation for extracting field data properties.

A set of climate-related indices that have been outlined elsewhere [13,38] was used here as metrics for imported vector and disease establishment risk. These rely on the assumption that the climatically-sensitive disease vectors require similar climatic conditions in their new locations to that which they experienced and survived in at their previous home locations in order to successfully establish. Moreover, for imported cases of vector-borne diseases to result in onward transmission in new locations, climatic conditions that promote vector activity, similar to the origin location where the disease was contracted, are required. Three simple indices were calculated:

1. Climatic Euclidean Distances (CEDs) are a measure of similarity in climatic regime between one location and another, and in this case were calculated through obtaining measures of rainfall (*r*), temperature (*t*) and humidity (*h*) for each airport location for each month from the gridded climatic datasets described previously. The CED between airport i and j was then calculated by $1/\surd \left({\left(r_{i}-r_{j}\right)}^{2}+{\left(t_{i}-t_{j}\right)}^{2}+{\left(h_{i}-h_{j}\right)}^{2}\right)$[13,38].

2. Climatic Euclidean Distance scaled by passenger volumes (or traffic, *t*) (CEDt), provides a more relevant relative measure of insect/disease introduction risk and consequent establishment/spread by route, through taking into account not only climatic suitability between regions, but the level of traffic on connecting flight routes. CEDt is calculated as in CED above, but the resulting values are normalized to the 0–1 range and multiplied by the traffic levels on the route in question, which have been normalized through dividing by the maximum traffic value in the database [13,38].

3. Climatic Euclidean Distance scaled by passenger volumes and 'risk' in terms of predicted disease endemicity or probability of vector presence (CEDtr) at the flight origin, provides a more relevant relative measure of insect/disease introduction risk to non-endemic/vector-free regions and consequent establishment/spread by route, through accounting for not only volume of traffic, but disease/vector prevalence at origin locations. CEDtr is calculated as in CED above, but the resulting value is normalized and multiplied by the normalized traffic levels on the route in question and by predicted disease endemicity or vector presence probability at the origin location, again normalized to the 0–1 range [13,38].

### Risk assessments

Three specific ‘risk assessment’ groups of functions have been built into the VBD-AIR tool and the following paragraphs describe the rationale and content of each of these assessments, provide examples on getting user specified outputs and documents the caveats and limitations of each assessment.

Imported vector-borne disease case risk assessments are aimed at providing estimates for the relative risks between scheduled flights of incoming air passengers carrying cases of the user selected disease. Two simple measures are calculated for the selected airport, disease and month: (i) scheduled passenger capacity for 2011 on all routes coming from endemic or outbreak risk regions of the selected disease; (ii) these passenger capacity numbers normalized through dividing by the maximum traffic value in the database and rescaled by the disease risk value in the region of origin. The first metric provides a simple measure of the maximum number of passengers arriving each month from areas of the world where transmission of the selected disease is either known to be endemic, predicted to occur or has occurred in the past. Comparing these between routes and months provides a first pass measure of risk route and timing prioritization. The second one provides a refinement to the simple measure in (i) that incorporates information on disease endemicity (on a 0–1 scale) at the origin.

The risk values calculated are based solely on scheduled incoming flight routes in 2011, the traffic capacity on these routes and the estimated endemic disease risk at the origin airport region. These estimates do not take into account additional risk-modifying factors such as actual passenger numbers, traveler activities and prophylaxis use, seasonal variations in disease transmission, chartered flights or multiple stopovers. Further details can be found in the VBD-AIR user guide and the user-generated reports available from the online tool (http://www.vbd-air.com).

Onward transmission risk assessments are aimed at providing basic estimates for the relative risks between scheduled routes of incoming flights bringing infected passengers, and those passengers coming into contact with active, competent vectors to facilitate onward transmission. Two simple measures are calculated for the user-selected airport, disease and month: (i) Flight capacities rescaled by climatic similarity between origin and destination regions for flights originating in disease endemic or outbreak prone regions (CEDt). This metric is based on the assumption that the risk of infected passenger arrival and onward disease spread is related to the amount of traffic between locations (increasing the probability of disease carriage) and also the similarity of the climate at the destination to that of the origin, since vector activity is required at both locations to firstly provide infected passengers, and secondly prompt onward transmission at the selected destination. (ii) The previous metric rescaled by the disease endemicity/risk value, r, at the origin region (CEDtr) [4,24]. This provides additional refinement of the previous metric to account for spatial variations in disease risk across the world. Finally, the opportunity to overlay a map depicting travel time to the nearest major settlement is available, to provide contextual information on (i) airport disease accessibility at the origin and (ii) the potential ease of disease spread upon arrival.

The risk values calculated are based on scheduled incoming flight routes in 2011, the traffic capacity on these routes, the climatic similarity to origin regions and the predicted presence of the disease at the origin airport region and competent vector at the destination airport region. These estimates do not take into account additional risk-modifying factors such as passenger numbers, vector preferences, passenger activities, seasonal variations in disease vector population sizes, or vector control measures in place.

Imported vector risk assessments is aimed at providing basic estimates for the relative risks between scheduled routes of incoming flights bringing exotic disease vectors and their consequent establishment. Two simple measures are calculated for the user-selected airport, vector and month: (i) Flight capacities rescaled by climatic similarity between origin and destination regions for flights originating in vector presence regions. This metric is based on the assumption that the risk of exotic vector arrival and establishment is related to the amount of traffic between locations (increasing the probability of carriage) and also the similarity of the climate at the destination to that in its home range, accounting for seasonal variations. (ii) The previous metric rescaled by the vector suitability value, r, at the origin region (CEDtr) [3,4,24]. This provides additional refinement of the previous metric to account for spatial variations in vector suitability and abundance across the world.

The risk values calculated are based on scheduled incoming flight routes in 2011, the traffic capacity on these routes, the climatic similarity to origin regions and the predicted presence of the disease at the origin airport region and competent vector at the destination airport region. These estimates do not take into account additional risk-modifying factors such as passenger numbers, vector preferences, passenger activities, seasonal variations in disease vector population sizes, or vector control measures in place.

### VBD-AIR tool architecture

**Figure 3:**
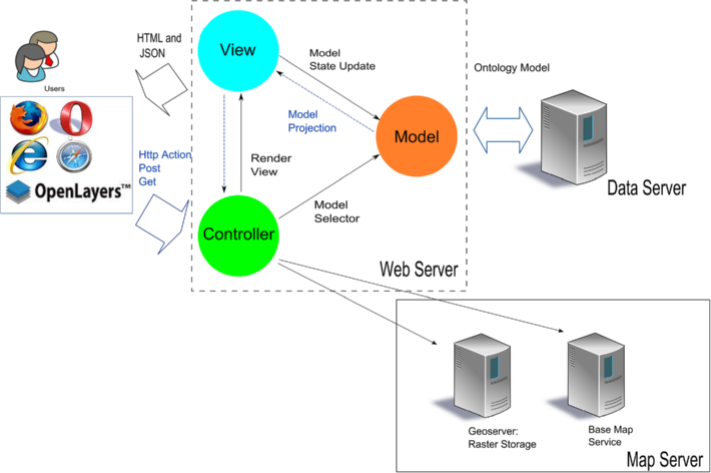
**The architecture of the VBD-AIR tool.** VBD-AIR adopts a three tier design: Web Server Tier, Data Server Tier and a Map Service Tier. The web interface follows AJAX standards. The web server implements a Model-View-Controller (MVC) framework. The data server is an SQL database, employed in a snow-flake structure and interpolated in the web server as data entities. The Web Mapping Services hosts the coverage file for predicted disease and vector distributions on a GeoServer. The base map service retrieves maps from Google and OpenStreetMap.

VBD-AIR adopts a three tier design: (i) a web server tier, (ii) a data server tier and (iii) a map service tier (Figure3). The web server for VBD-AIR is constructed in an asp.net Model-View-Controller (MVC) framework adopting agile development principles. The MVC framework provides facilities for separating the modules and functionalities defined by the knowledge integration method via the MVC framework [39]: the model reacts and responds to the commands from the controller, queries the database and transfers data to the application domain (usually a data view is generated), and alters the application state if instructions are received from the controller. The view formulates a web form with structural data presentation to the user. The controller receives inputs from the users via html form and sends it to the model.

In the VBD-AIR web server (Figure3), the controller is responsible for the page actions from the user (also known as 'Http Post' and 'Http Get'), as the user clicks on a button or a dropdown list, the collection of the user’s operations are sent as an object to the server. The controller receives actions from the user and selects an appropriate model for building a data view. The data view, which is usually a projection of a data model, is rendered in an HTML form or a JSON (JavaScript Object Notation) format for data exchange between the server and the client. In VBD-AIR particular, the knowledge graph is rendered in JSON. The model in VBD-AIR is a repository of intermediate data, which is a collection of data entities and their relationships generated from the data server. This data assimilation process includes an object-oriented mapping procedure and add-ons of analytical logics. The controller also has the responsibilities to call the map server for the overlays of base maps and raster spatial dataset layers.

The data server is an SQL database, employed in a snow-flake structure, as the airport table is assigned as a fact table in the database. This table contains geographical information on the 3,682 airports. All disease, vector, route capacity and climate spatial data are mapped into this relational database management system. The data have been rectified for occasional misalignment error using ArcGIS v10. This schema simplifies the data structure for queries on airports and facilitates the data exchange process and interoperation.

The communication between the web server and the data server is largely based on a repository pattern with a predefined data model. The repository pattern is a middleware container between the study objects and the data. It is capable of aggregating data collections using precompiled queries to the database [40]. In general, the repository pattern provides a simple querying structure for intermediate data and facilitates the data exchanges.

The map service is composed of two service providers: “base map services” and “web mapping services for raster spatial datasets”. The base map services provide an overlay of the world map from either Google Maps or OpenStreetMap, which illustrate the viewing extents for the significant air routes. Furthermore, it provides functions such as an auto zoom to the viewing extent when a map is generated or an airport is selected. The web mapping services for raster spatial datasets host the map files for disease and vector distributions on a GeoServer. This service is able to stream the raster dataset to the client’s browser via Web Mapping Services (WMS) and the user can then see the raster dataset overlay in their browser.

Once the user opens the VBD-AIR website (http://www.vbd-air.com), the Open layer library with preset visualization functions is loaded to the users’ browser. The base map is generated from the base map services and a raster dataset overlay is generated from the Geoserver. Feature layers for airports and air routes are populated from the JSON string streaming from the controller, and this JSON string is generated according to the user input on the html form of input choices, which contains airport information, disease/vector probabilities, flight passenger capacity information and climatic conditions. Finally, these feature layers are visualized and overlaid on the base map. Supported 'mouse events' include mouse-driven map navigation and automated air route re-rendering. Users can navigate the mouse pointer to an airport or an air route to obtain more detailed information and select different rendering scenarios on imported risks or onward transmission. When a metric is chosen, the routes are colored to distinguish between where the metric is larger than the average for all routes shown, and where it is smaller. The top 10 routes ranked by the user's chosen metric are also displayed.

As the development of VBD-AIR has and will continue to involve multidisciplinary effort, we have implemented a test-driven design routine to utilize the MVC framework, so as to minimize the cost for communication. Test Driven Development (TDD) permits flexibility for a more feature driven development. A feature can be treated as a separable function in a project [41]. TDD helps in separating the concerns of various implementations of features, for instance, the addressing of raster data overlay and PDF report generation should rely on different programming libraries, and thus, they have totally different input and output. TDD can state the objectives of these two features by dependency injection, which uses an interface as a contract to declare functionalities. The contracting interfaces could be substituted with workable classes to implement the functionality. In the implementation, different programming resources can be imported to fulfil the requirements. TDD enables the design of reusable features that can be used across multiple development efforts.

## Results

**Figure 4:**
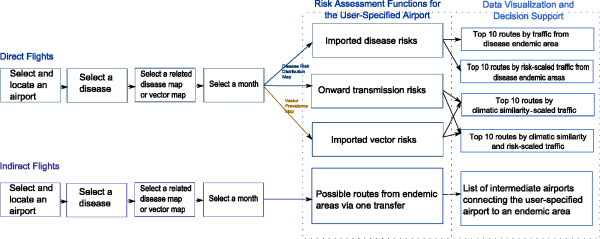
**Flow of user input for the VBD-AIR tool.** Risk assessment functions for the VBD-AIR tool. “Imported disease risks”, “onward transmission risks” and “imported vector risks” are provided for the direct flight route scenario and the possible routes from endemic area via one transfer are provided for the indirect flight scenario.

The test version of VBD-AIR is available at http://www.vbd-air.com. In the current version, VBD-AIR enables users to explore the interrelationships between the global distributions of the four major vector‒borne infectious diseases, seasonal climatic changes and seasonally changing air traffic capacities, and the full set of user inputs available in outlined in Figure4.

**Figure 5:**
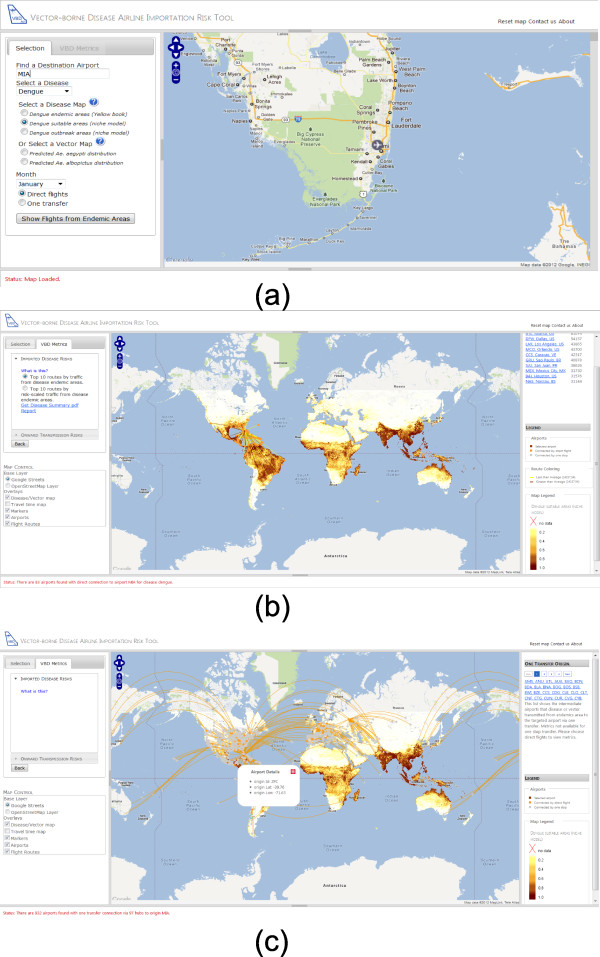
**User input example for the VBD-AIR tool.** A sample user input for the VBD-AIR tool for a user interested in imported dengue infection risks to Miami in January. **a**) The user selects their airport, disease and month of interest; **b**) The result shows the direct flight routes from dengue suitable areas to Miami, and the user can select risk assessments that include imported disease risk and onward transmission risks; **c**) The result shows the direct and one-transfer flight routes from dengue suitable areas to Miami, and the user can navigate through all the results for more detailed information.

The visualization of the air travel network within VBD-AIR follows Schneiderman’s principle for data visualization: Overview first, zoom and filter, then details-on-demand [42]. The web interface provides a map view of the flight connections from endemic disease or vector presence areas to a user-selected airport, with a predicted disease risk or vector presence map overlaid (Figure5). The user can view and navigate through the flight routes from each of the airports within the endemic or vector presence regions. As the user selects their choice of risk assessment type (imported disease risks, onward transmission risks or imported vector risks – see Figure4), the view supports an automatic colouring scheme to render the origin airports and the routes based on the user input and the objectives for risk assessment. Moreover, when the user clicks on an airport, detailed location information for that airport is provided. The interface is also well equipped with a comprehensive user guide, a short tutorial and integrated pop-up help windows so that the user can get a better understanding of the tool functionality, datasets and outputs.

For both the “direct flight” and “indirect flight” options (Figure4), users are required to input the name or city of their airport of interest, disease of interest, and their related choice of disease distribution or vector presence map. The direct flight options provide “imported disease risks” and “onward transmission risks” functions if the user selects a disease map, and “imported vector risks” if the user selects a vector map. The imported disease risk option provides the top 10 routes by route capacity from disease endemic areas and the top 10 routes by risk-scaled traffic from disease endemic areas. The onward transmission risks and imported vector risks provide top 10 routes by climatic similarity scaled traffic and top 10 routes by climate similarity and disease/vector risk scaled traffic. The option to produce a more detailed PDF report output is provided to enable users to examine output statistics in greater detail and view longitudinal data. An option is also available to view indirect flights to the airport of interest, which highlights the possible routes from disease endemic areas via a single flight transfer, and a list of intermediate airports.

A sample user input is presented in Figure5. In this example, the user is interested in the risks of dengue importation to Miami airport in January by direct flights. Once the user selects Miami airport through the auto-complete text box, the map zooms to show the region (Figure5a). The user then selects dengue from the list of diseases available and chooses the spatial representation of dengue risk they are interested in (see Data section above for information on each map), and can click on the question mark icon to get help in choosing, which in turn links to the user guide to obtain further information. The user then selects January from the drop-down month list and chooses direct flights, before choosing to display their selections. The next screen (Figure5b) shows the user’s chosen dengue risk map with all direct flights from endemic areas to Miami overlaid and colored by whether the traffic on the route is greater or less than the average traffic on all routes to Miami from endemic areas. The user can then select metrics from the imported disease risks box in the top left, choosing whether to view the top 10 routes by traffic from endemic areas or by traffic scaled by disease risks at the origin location. In each case, the top 10 risk routes are displayed in the box in the top-right. For more detailed quantification of imported disease risks metrics, the user has the option to view a customized PDF format report. Finally, the user can view all those flights that are connected to Miami from endemic areas by a single flight change through returning to the data selection screen and selecting the ‘one transfer’ option (Figure5c).

## Discussion

With no apparent end in sight to the continued growth in global air travel, we must expect the continued appearance of disease-spreading vector invasions and vector-borne disease movement. Approaches that can inform decision makers on the risk factors behind these importations and onward spread risks can be used to focus surveillance and control efforts more efficiently. This paper and the VBD-AIR tool it describes show that multiple datasets on many aspects relating to the risk of movement of insect vectors and vector-borne diseases through the global air network can be compiled to provide such information through a user-friendly web tool. The principal function of the VBD-AIR tool is to provide an evidence base for assessing the role of air travel in the spread of vector-borne diseases and their vectors through available spatial data. The VBD-AIR tool is designed with a wide range of potential users in mind. These include planners and decisions makers based in state and local government, in particular, those at international and domestic airports tasked with planning for and dealing with health risks and allocating limited resources. It is clear from exploration of outputs from VBD-AIR that each region, airport and flight route has a differing risk profile in terms of disease and vector importation, determined largely by the structure of the air network and its congruence with infectious disease distributions and outbreaks and vector distributions and seasonality, yet this is rarely quantified and used when control methods and surveillance are considered.

The VBD-AIR tool shows that a multi-disciplinary approach, which draws on a variety of spatial data on factors known to influence the spread of vectors and the diseases they carry, offers potential for assessing the risk of disease importation. A range of uncertainties and limitations do still exist in the datasets and outputs presented however, and users are made aware of these within the full user guide and throughout the information boxes within the tool. Firstly, VBD-AIR considers only direct flights and their capacities within metric calculations, rather than actual passenger numbers or stopovers, and users are made aware of the uncertainties that this entails [43]. Within the disease and vector distribution modeling processes, uncertainties are inherent throughout [44], particularly in those regions with little field data to inform predictions. Moreover, we have treated the vector distributions as single homogenous types of mosquito, yet competition, competence, adaptation and preferences can vary widely across their global distributions [20-22,33]. Accurate data on outbreak locations and sizes, as with many diseases, are also difficult to obtain to be sure of comprehensive assessments of risk, however, improvements in global surveillance and the rapid availability of data are improving (e.g.[45]). Also, the distance between two airports and the population size of these airports have not been explicitly incorporated in the risk assessments here. Proximity to endemic area plays an important role in vector-borne disease importation [46], while city population size can be utilized to estimate the rate of disease movement between pairs of airports [47]. Further the use of climatic similarity measures may not be appropriate to certain contexts, such as those where arid climatic conditions prompt increased water storage, leading to rises in vector-borne disease risks, rather than the decreases that may be indicated by CEDs. Finally, how to interpret and act upon the kind of relative risks identified in VBD-AIR is a challenge yet to be overcome, but various approaches to mitigating risks are presented within the tool text boxes and user guide [3].

Future updates are planned to VBD-AIR that will expand its capabilities, as the MVC framework is designed to be flexible for robust expansion. These will include: (i) Regular updates of the disease and vector distribution maps, as new survey data and outbreak reports become available; (ii) updates to the flight data as new information on flight capacities and routes becomes available; (iii) additional scenario-related functionality will be built into the tool based on a set of control and mitigation options. This will provide users with guidance on approaches to limiting imported cases and vectors, and mitigating the effects of vector establishment or onward disease transmission; (iv) the interactive incorporation of the accessibility datasets. At present this represents a simple visualization of access to provide context, and upcoming extensions will focus on building in measures of access to better capture airport catchment areas and estimate likely regions impacted by imported cases or vectors; (v) the incorporation of extra vector-borne diseases and vectors. The choice of additional diseases and vectors will depend upon availability of sufficient spatial data for mapping or validated global maps. Candidate diseases include leishmaniasis, Rift Valley fever and chagas disease.

It is envisioned that future research beyond the simple updates and tool expansions described above will build upon VBD-AIR to continue to improve quantification of these aspects, drawing on newly-developed spatial datasets and mathematical models of transmission, to provide an evidence base to enable airports, airlines, and public health officials to assess the appropriateness and efficacy of current control, surveillance and treatment practices, and tailor strategies to these differing risk profiles for each disease, route and airport. Three specific areas of research should be examined:

(i)  Constructing geospatial information databases on global endemic disease distributions, and building a framework for the rapid inclusion of outbreak reporting data from surveillance databases such as Health Map (http://www.healthmap.org) [41]. Increasingly, spatial information on the prevalence of directly-transmitted and insect-borne diseases are being made available, and approaches for using these data to build distribution maps and dynamic transmission models are following. The potential of combining such data with air traffic data for forecasting disease movements has been shown for a handful of diseases in specific locations, but this potential has yet to be realized at global scales.

(ii)  Increasing the sophistication of flight passenger movement data and models. Existing models of disease movement over air networks are generally driven by flight capacity and direct flight data [48], missing valuable information on stopovers, actual passenger numbers and lengths of stay. Sample datasets on ticket sales and flight occupancy (e.g. US Transtats T100 and DB1B data http://www.transtats.bts.gov ) should be utilized to derive models that can better replicate realistic passenger flows, for integration with the disease risk data [43]. Also, the incorporate of proximity measure (such as geographical distance or flight time between pairs of airports) and population information which airports serve is likely to facilitate the estimations of the actual travel flows between two airports [46,47].

(iii)  The development of stochastic analogues of existing deterministic approaches to modelling of disease movement through air networks that are capable of handling input parameter distributions rather than simple mean values, and provide measures of uncertainty with output forecasts. The process of disease importation is a stochastic process, and, depending upon the disease, each relevant variable (e.g. seasonal variations in transmission, passenger numbers, and infection risk) can exhibit substantial variations from the mean and include uncertainty in the way they are measured. By simulating risks of importation from literature-derived probability distributions for each variable, improved and more informative model outputs could be produced that would enable the user to better understand and manage the uncertainties inherent in forecasts (e.g. [49]).

## Conclusions

Increases in global travel are happening simultaneously with many other processes that favour the emergence of disease [50,51]. Air travel is a potent force in disease emergence and spread, and the speed and complexity of modern aviation makes both geographical space and the traditional ‘drawbridge’ strategy of disease control and quarantine increasingly irrelevant [6]. With no apparent end in sight to the continued growth in global air travel, we must expect the continued appearance of disease vector invasions and vector-borne disease movement. Approaches that can inform decision makers on the risk factors behind vector-borne disease importation and onward spread risk can be used to focus surveillance and control efforts more efficiently. The VBD-AIR tool shows that multiple datasets on many aspects relating to the risk of movement of vector-borne diseases and their vectors through the global air network can be compiled to provide such information. VBD-AIR is available at http://www.vbd-air.com.

## Abbreviations

CED: Climatic euclidean distance; CEDt: Inverse climatic euclidean distance scaled by passenger volumes (or traffic *t*); CEDtr: Inverse climatic euclidean distance scaled by passenger volumes and 'risk' in terms of predicted disease endemicity or probability of vector presence; GIS: Geographic information system; HTML: Hyper text markup language; IATA: International air transport association; JSON: Javascript object notation; MAP: Malaria atlas project; MVC: Model-view-controller; OAG: Official airline guide; SQL: Structured query language; TDD: Test driven development; VBD-AIR: Vector-borne disease airline importation risk tool.

## Competing interests

The authors declare no competing interests.

## Authors' contributions

ZH, AD, YQ, and AJT worked on the concept, design, and implementation of VBD-AIR. ZH, AD, YQ built the architecture for VBD-AIR and implemented the web GIS. ZH, AD and AJT wrote the manuscript. All authors read and approved the final manuscript.

## Supplementary Material

Additional file 1 **This document shows the supplementary figures referred to in the main article. **The contents are as follows:a. Malaria maps, b. Dengue maps, c. Yellow fever maps ,d. Chikungunya map, e. Vector maps, f. Data flow in the VBD-AIR tool, g. Global travel time map. Click here for file
