# Supplementary material for: Web-based GIS: the vector-borne disease airline importation risk (VBD-AIR) tool
Source: Int J Health Geogr. 2012 Aug 14;11:33. doi: 10.1186/1476-072X-11-33 (PMC3503742; doi:10.1186/1476-072X-11-33)
Supplement: Additional file 1 — This document shows the supplementary figures referred to in the main article. The contents are as follows:a. Malaria maps, b. Dengue maps, c. Yellow fever maps ,d. Chikungunya map, e. Vector maps, f. Data flow in the VBD-AIR tool, g. Global travel time map. [file 1476-072X-11-33-S1.docx]

# Supplementary Information

This document shows the supplementary figures referred to in the main article. The contents are as follows:

a. Malaria maps

b. Dengue maps

c. Yellow fever maps

d. Chikungunya map

e. Vector maps

f. Data flow in the VBD-Air tool

g. Global travel time map

1. **Malaria maps**

i) *Plasmodium falciparum* endemicity in 2010


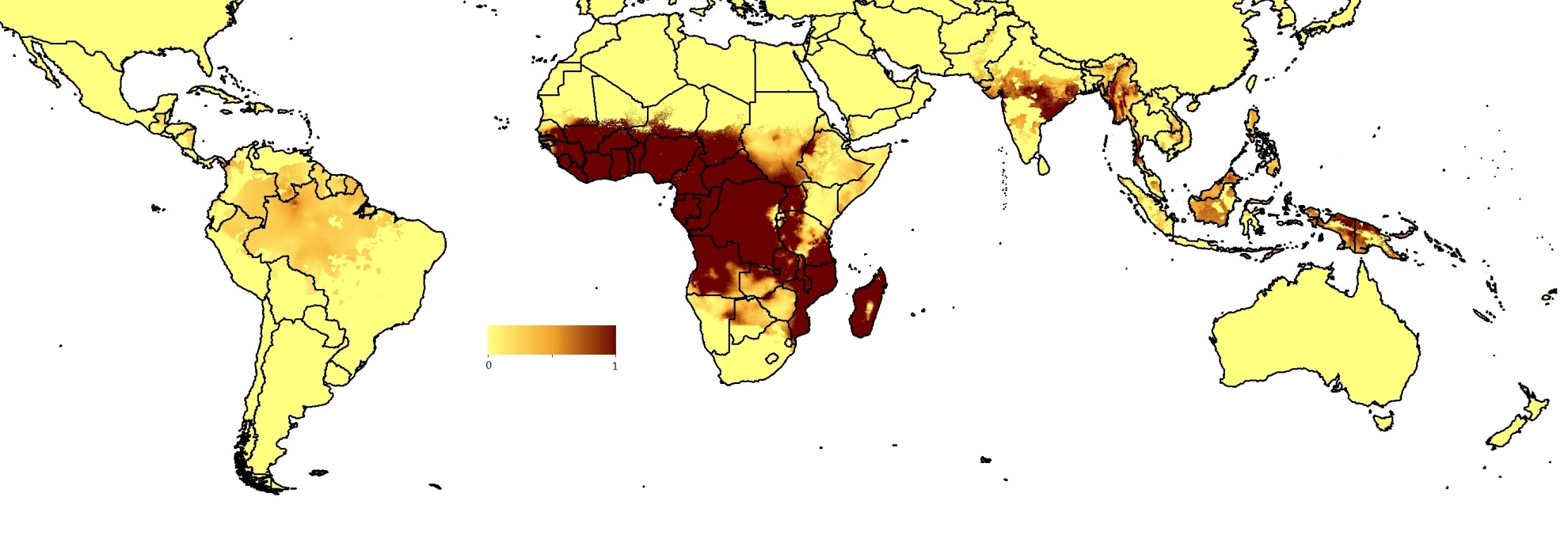


The Malaria Atlas Project (www.map.ox.ac.uk) recently produced a global map of *P. falciparum* malaria endemicity in 2010, based on over 24,000 community prevalence surveys. The methods used behind construction of this dataset are described in more detail here: <http://www.plosmedicine.org/article/info:doi/10.1371/journal.pmed.1000048>

ii) *Plasmodium vivax* limits of transmission in 2009


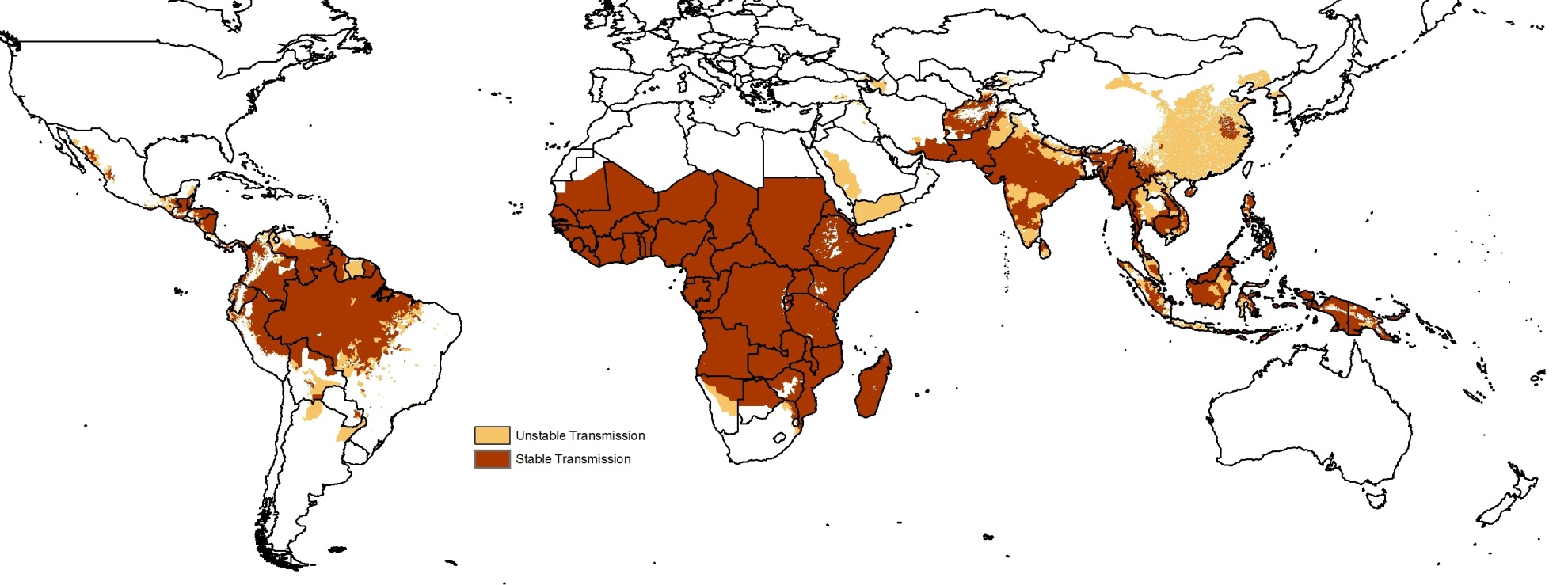


The Malaria Atlas Project (www.map.ox.ac.uk) recently produced a global map of the limits of *P. vivax* malaria transmission in 2009, based on thousands of reports of cases. The methods used behind construction of this dataset are described in more detail here: [www.plosntds.org/article/info:doi/10.1371/journal.pntd.0000774](http://www.plosntds.org/article/info:doi/10.1371/journal.pntd.0000774)

**b. Dengue Maps**

i) Dengue suitability map


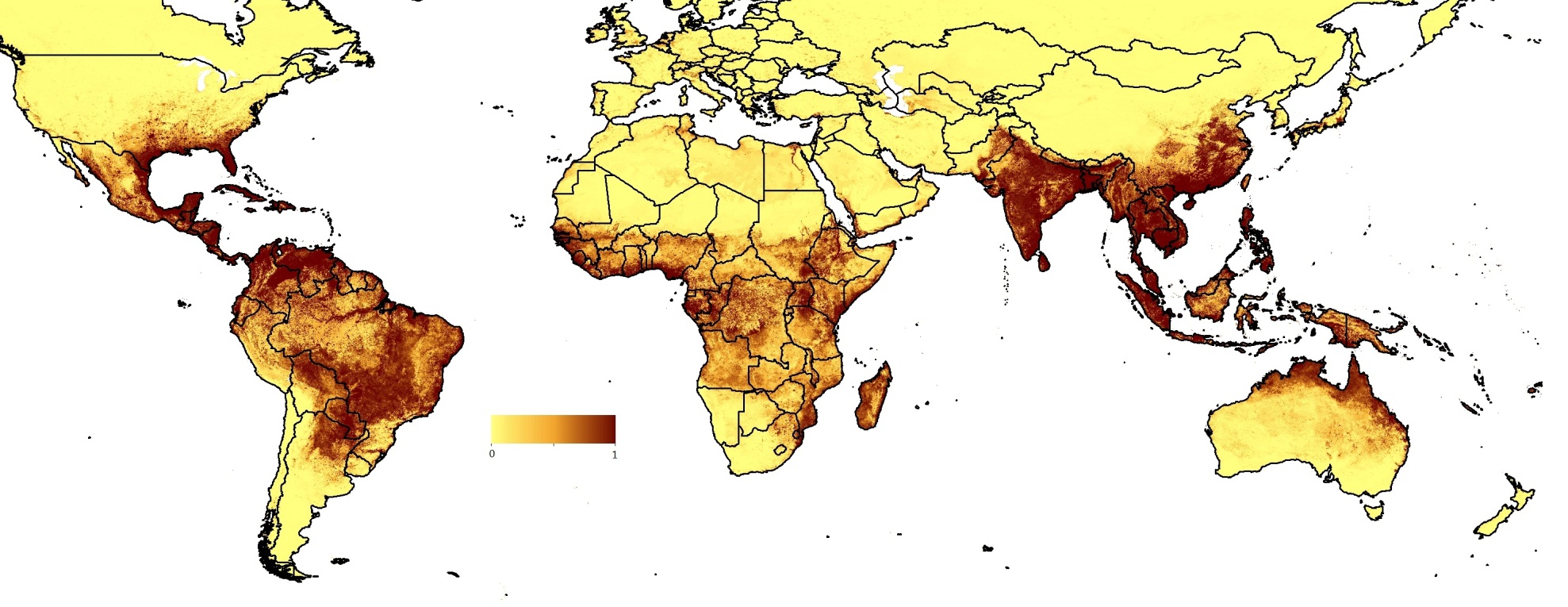


The predicted suitability for dengue transmission based on thousands of reports of dengue cases combined with environmental covariates in a boosted regression tree mapping framework.

ii) Dengue suitability in areas of known outbreaks in 2010


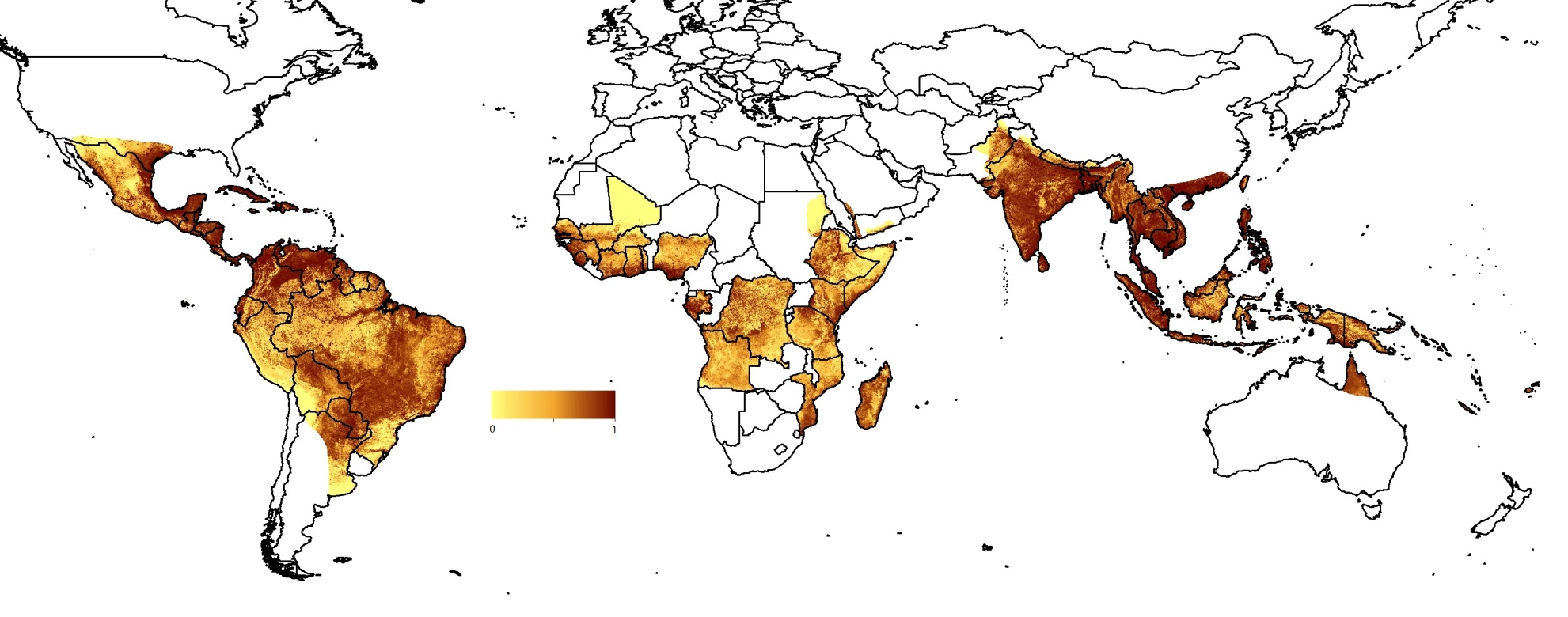


The predicted suitability for dengue transmission in areas of known outbreaks in 2010. This map is a refinement of the dengue suitability map in i) focusing solely on regions of recent confirmed transmission. It was masked so that only regions cited by the CDC Yellow Book as having transmission in 2010 were left.

iii) Dengue outbreak prone


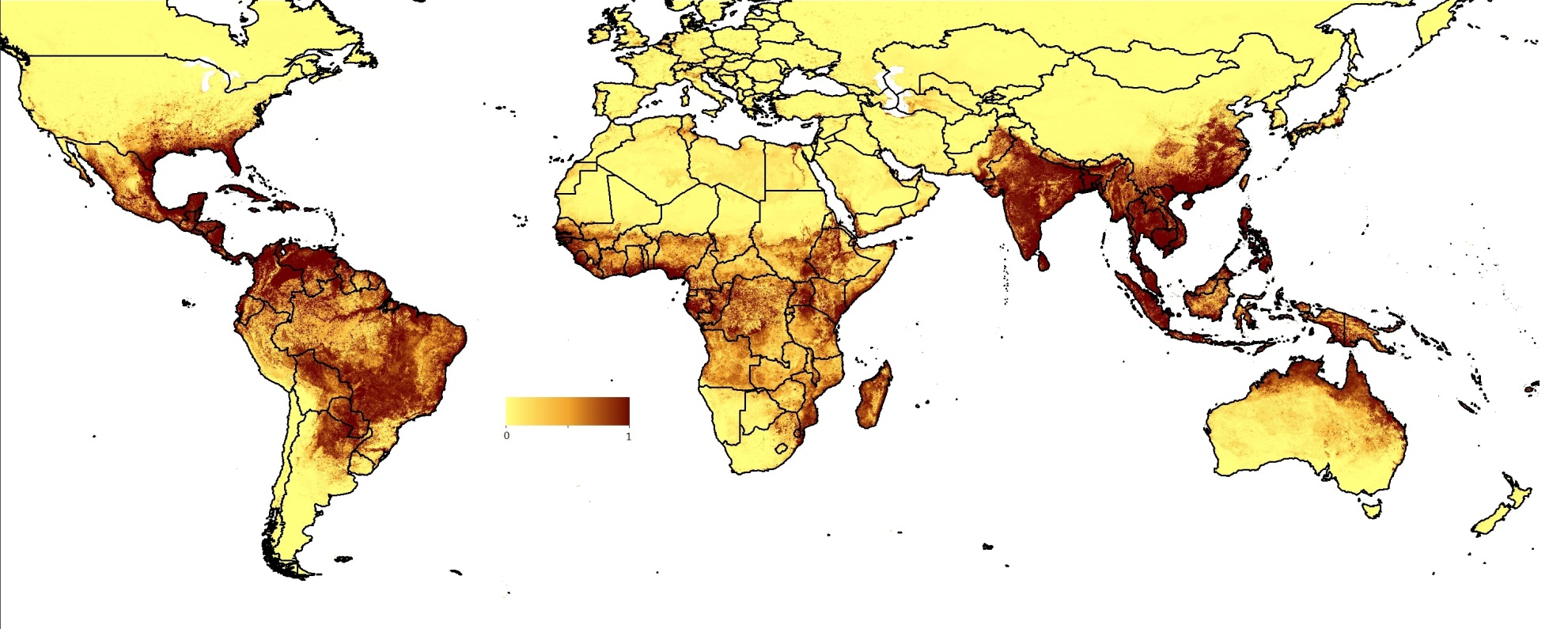


The predicted suitability for significant dengue outbreaks based on data on outbreaks since 2008 reported on Healthmap combined with environmental covariates in a boosted regression tree mapping framework.

**c. Yellow fever maps**

i) Yellow fever suitability


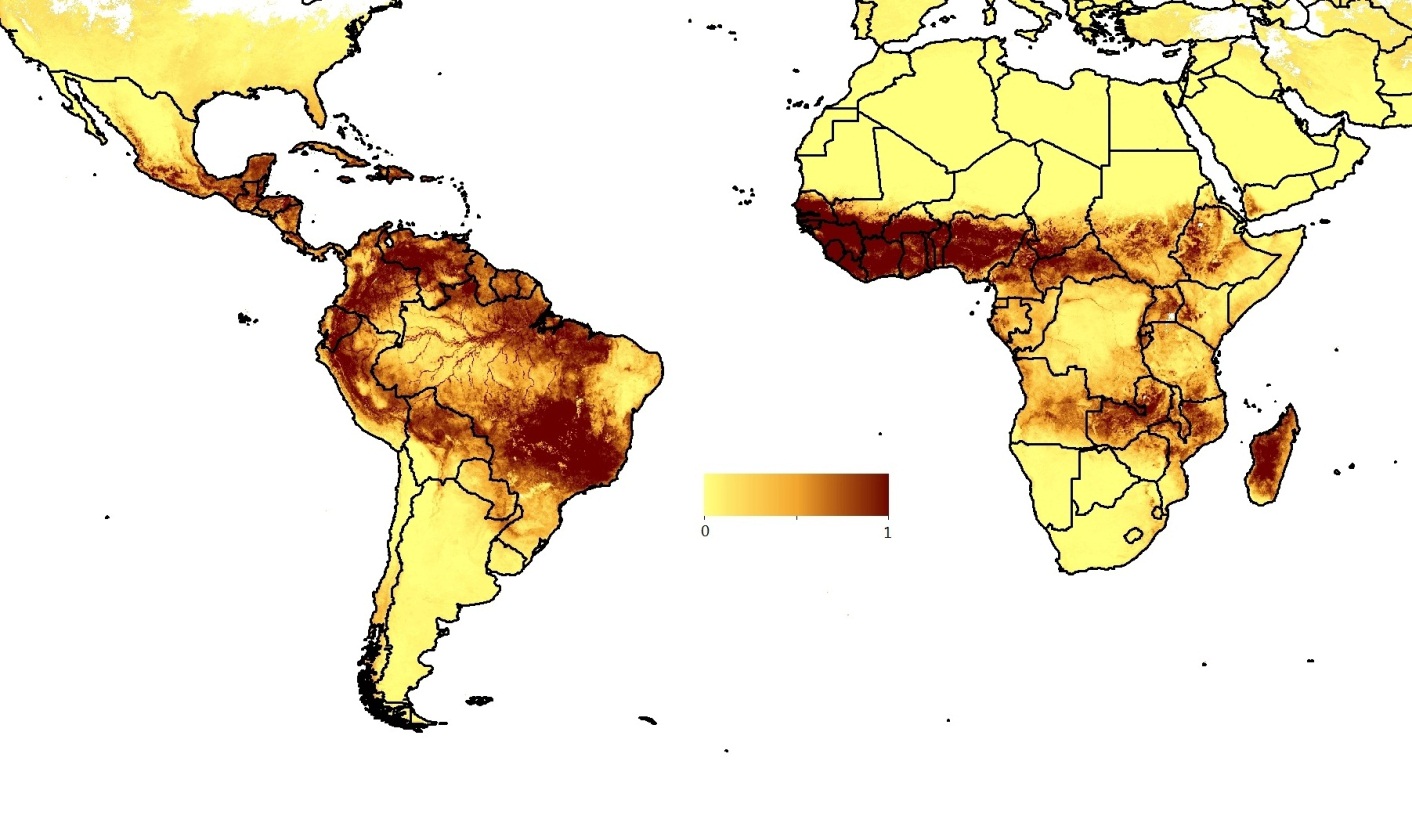


The predicted suitability for yellow fever transmission based on hundreds of reports of yellow fever cases combined with environmental covariates in a boosted regression tree mapping framework.

ii) Yellow fever outbreak prone


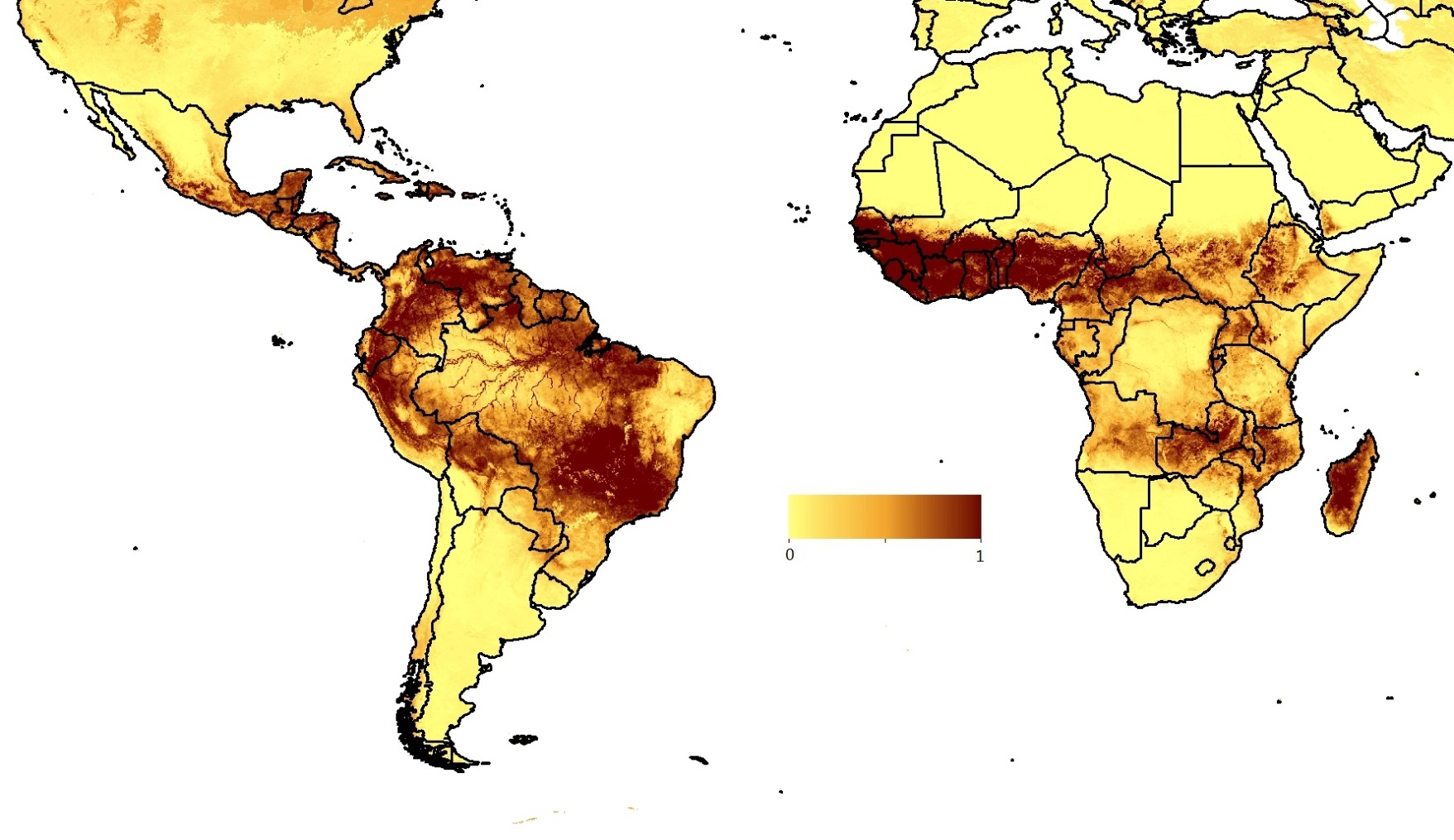


The predicted suitability for significant yellow fever outbreaks based on data on outbreaks since 2008 reported on HealthMap combined with environmental covariates in a boosted regression tree mapping framework.

**d. Chikungunya map**

i) Chikungunya outbreak prone


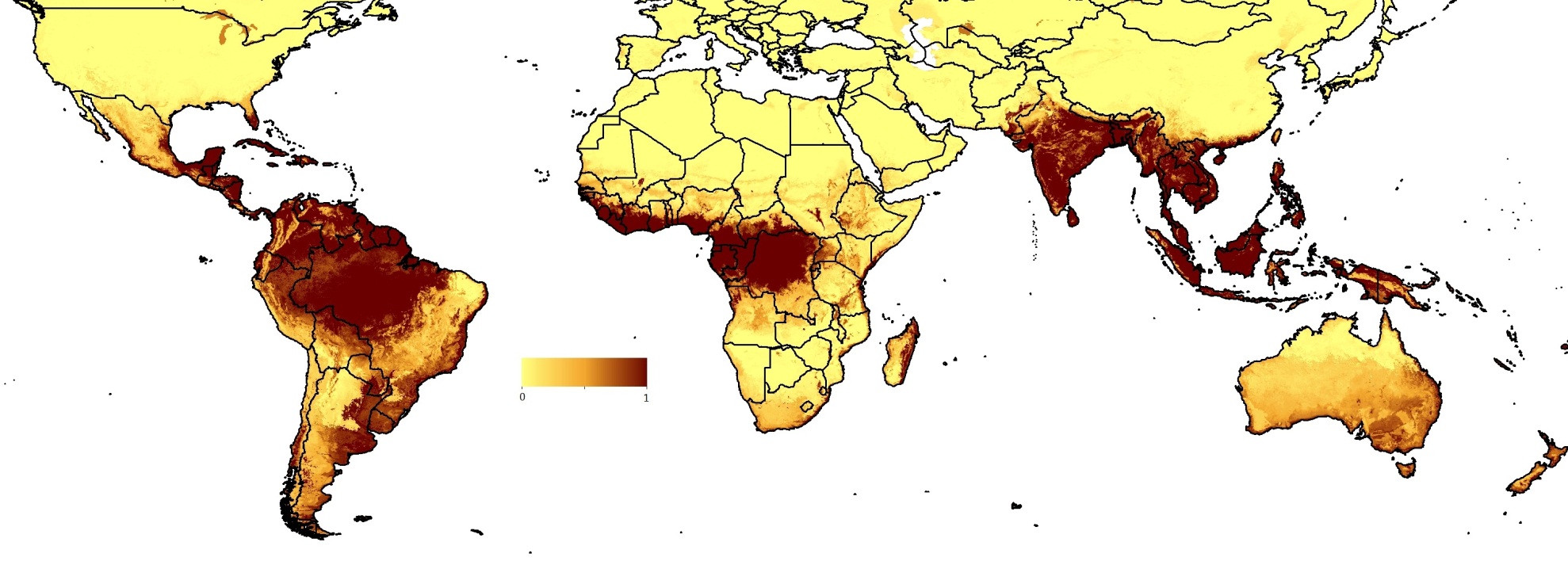


The predicted suitability for significant chikungunya outbreaks based on data on outbreaks since 2008 reported on Healthmap combined with environmental covariates in a boosted regression tree mapping framework.

**e. Vector Maps**

i) *Aedes aegypti*


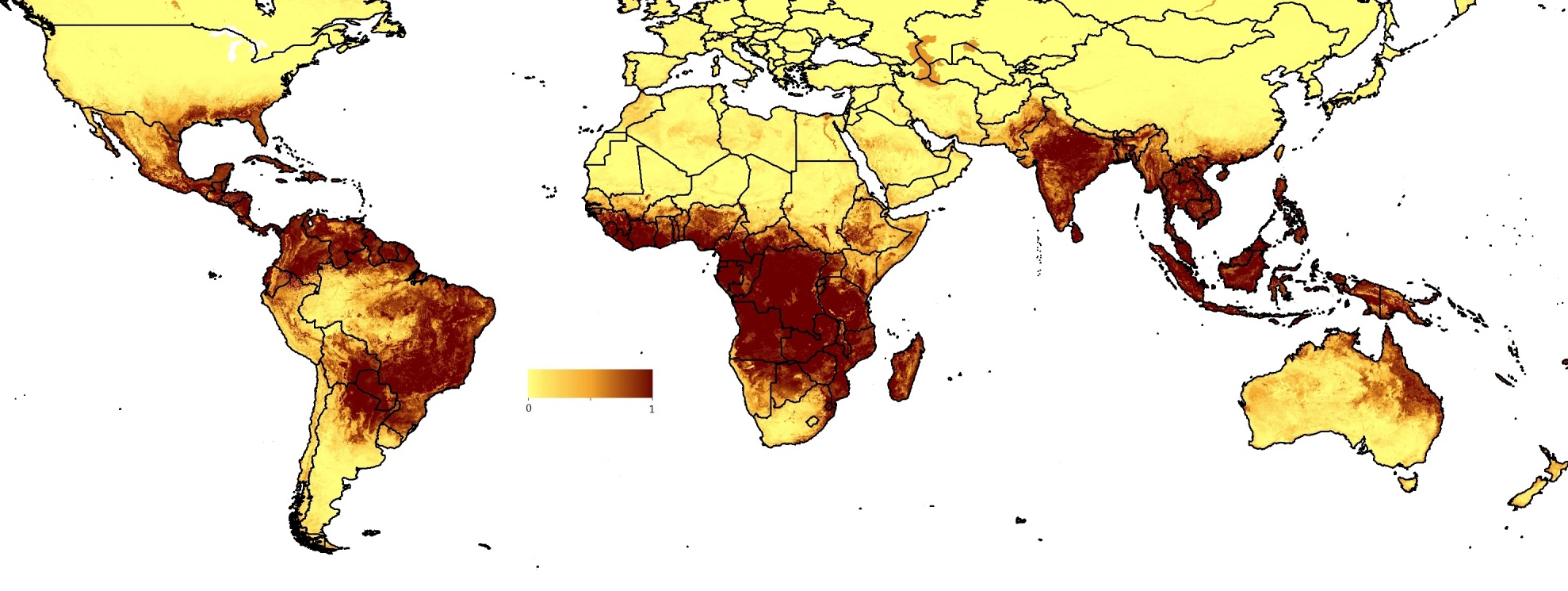


The predicted presence of *Aedes aegypti* based on hundreds of confirmed presence location data points. Hundreds of geographically‐located datapoints on field‐ caught *Ae aegypti* occurrence over the past 10 years were combined with climatic and environmental covariates within a regression tree mapping framework to produce a global map of predicted *Ae aegypti* presence.

ii) *Aedes albopictus*


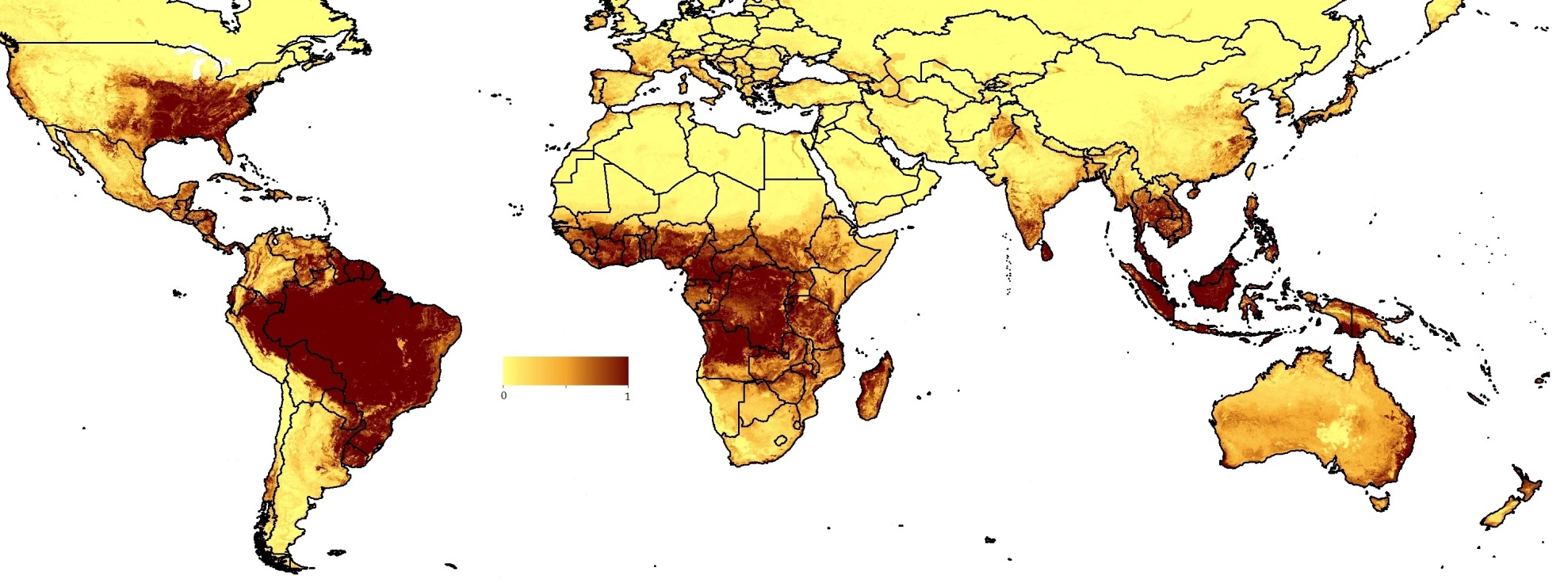


The predicted presence of *Aedes albopictus* based on hundreds of confirmed presence location data points. Hundreds of geographically‐located datapoints on field‐caught *Ae albopictus* occurrence over the past 10 years were combined with climatic and environmental covariates within a regression tree mapping framework to produce a global map of predicted *Ae albopictus* presence.

iii). *Anopheles*


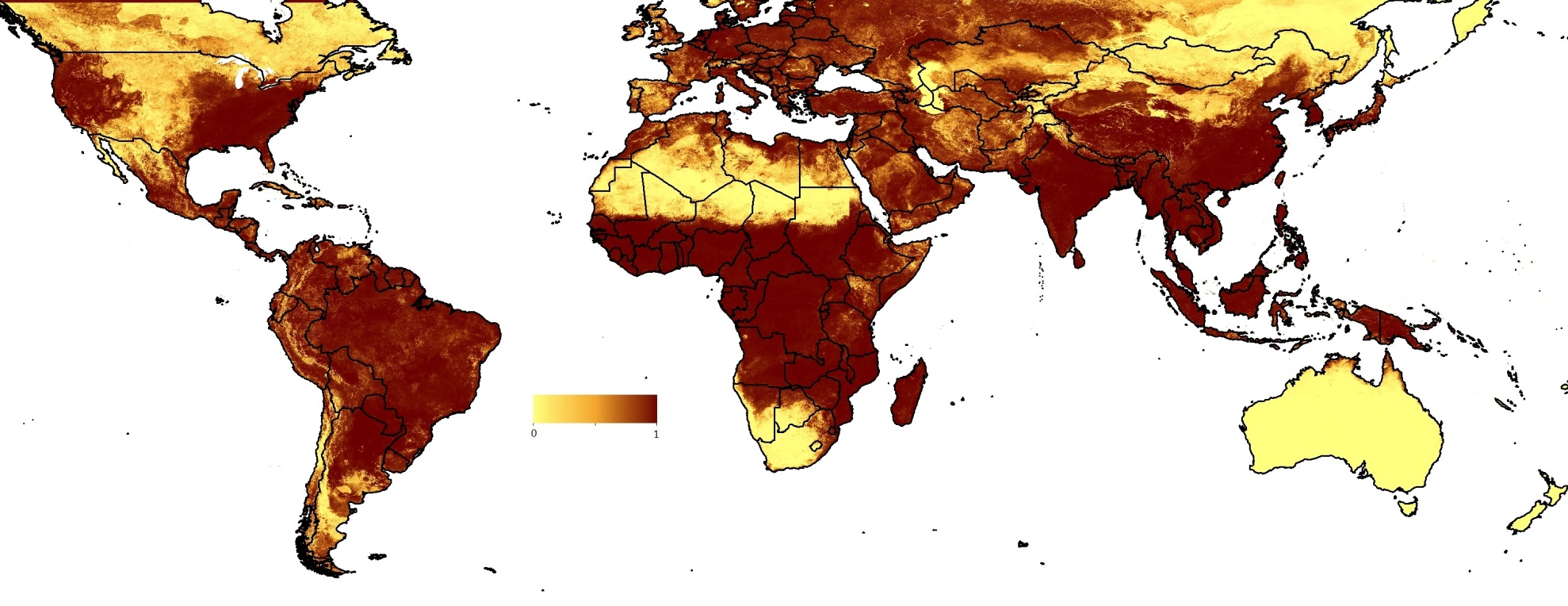


The predicted presence of one or more of the dominant *Anopheles* vectors of human malaria based on thousands of confirmed presence location data and expert opinion maps . Thousands of geographically‐references datapoints on the presence of the dominant *Anopheles* vectors of malaria have been gathered and used, in combination with environmental covariates, expert opinion maps and regression tree tools, to produce global maps of *Anopheles* distributions by the Malaria Atlas Project (www.map.ox.ac.uk). Here, these maps were combined to produce a global map of dominant malaria‐vector presence.

**f.Data flow in the VBD-Air tool**


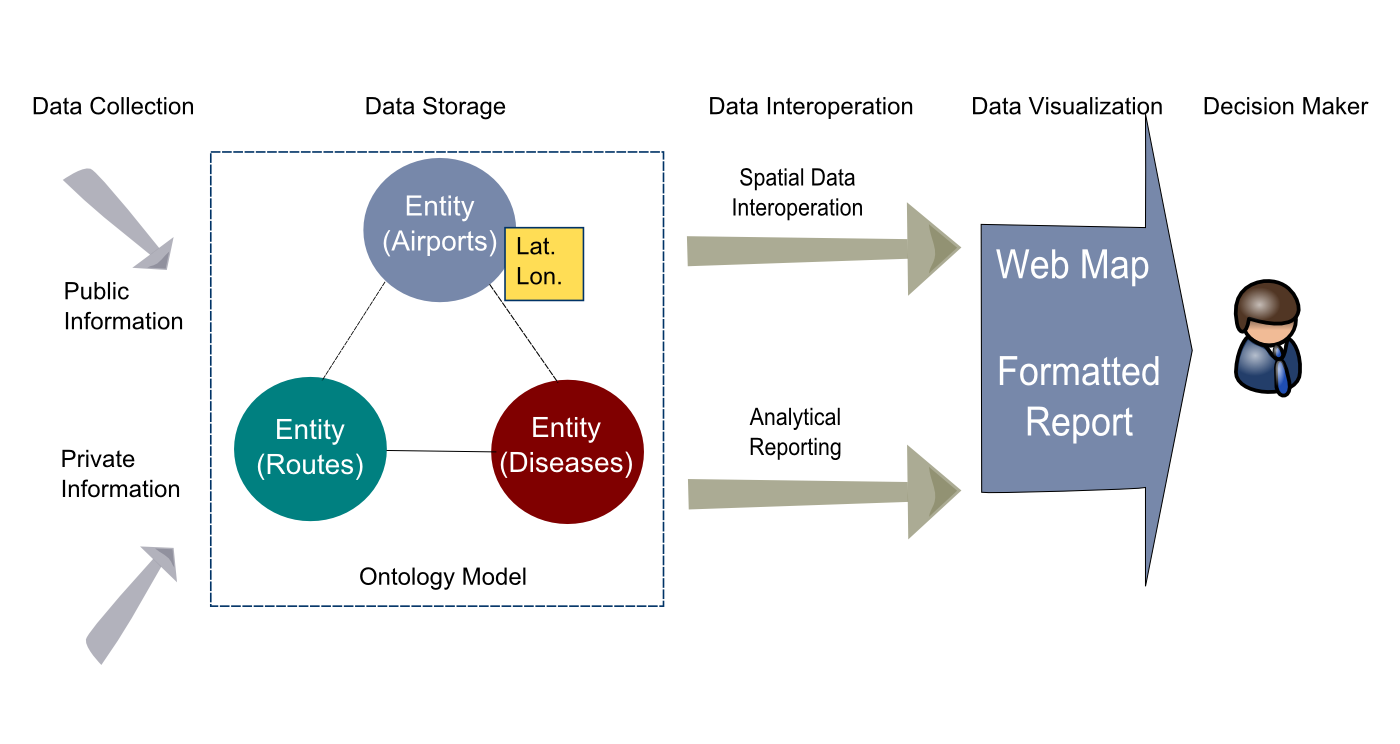


A data flow is presented here from raw data to decision maker. This data flow contains five stages: data collection allows organizations to gather information from various sources. Data storage constructs an ontology model for a knowledge graph connecting different concepts in the study domain; the location information is assigned to the vertices as attributes. Data interoperation establishes data channels for visualization. Data visualization provides interfaces to present spatial information on a web map or on a formatted report. The final objective of this data flow is decision making.

**g. Global travel time map**


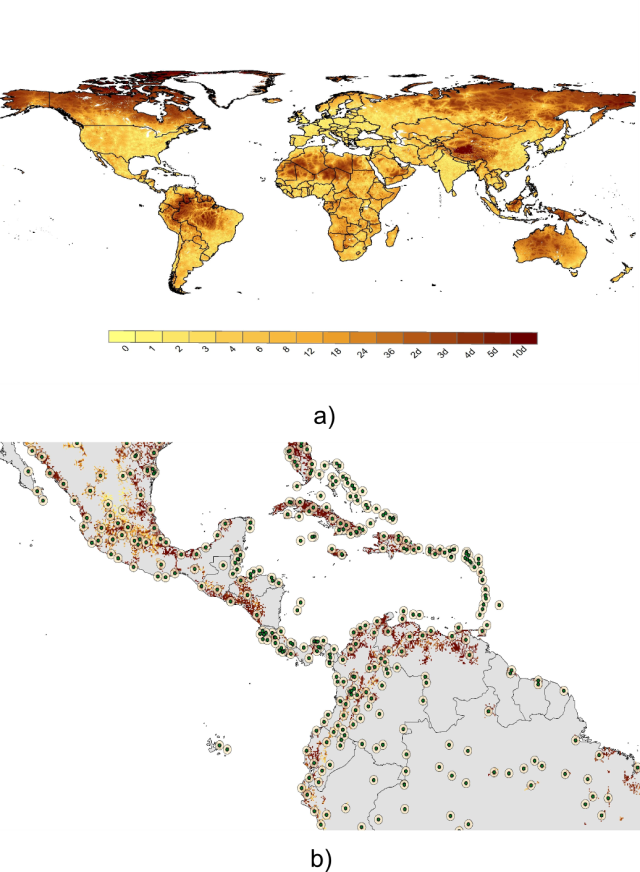


a) Travel time to the nearest major settlement (population size >50,000); b) Travel times of less than 2 hours to an airport for the central America region generated from the global map in (a). Each dot shows an airport location with a 50km buffer around it. These 2 hour and 50km thresholds were used to assign disease risks to airports (see main text).
